# Supplementary material for: Causal Relationship between Aging and Anorexia Nervosa: A White-Matter-Microstructure-Mediated Mendelian Randomization Analysis
Source: Biomedicines. 2024 Aug 16;12(8):1874. doi: 10.3390/biomedicines12081874 (PMC11351342; doi:10.3390/biomedicines12081874)
Supplement: Supplementary file 1 [file biomedicines-12-01874-s001.zip › Supplementary Figure/Figure S4/Singe SNP plot of MR analysis for TL to ubm-b-1765.pdf]

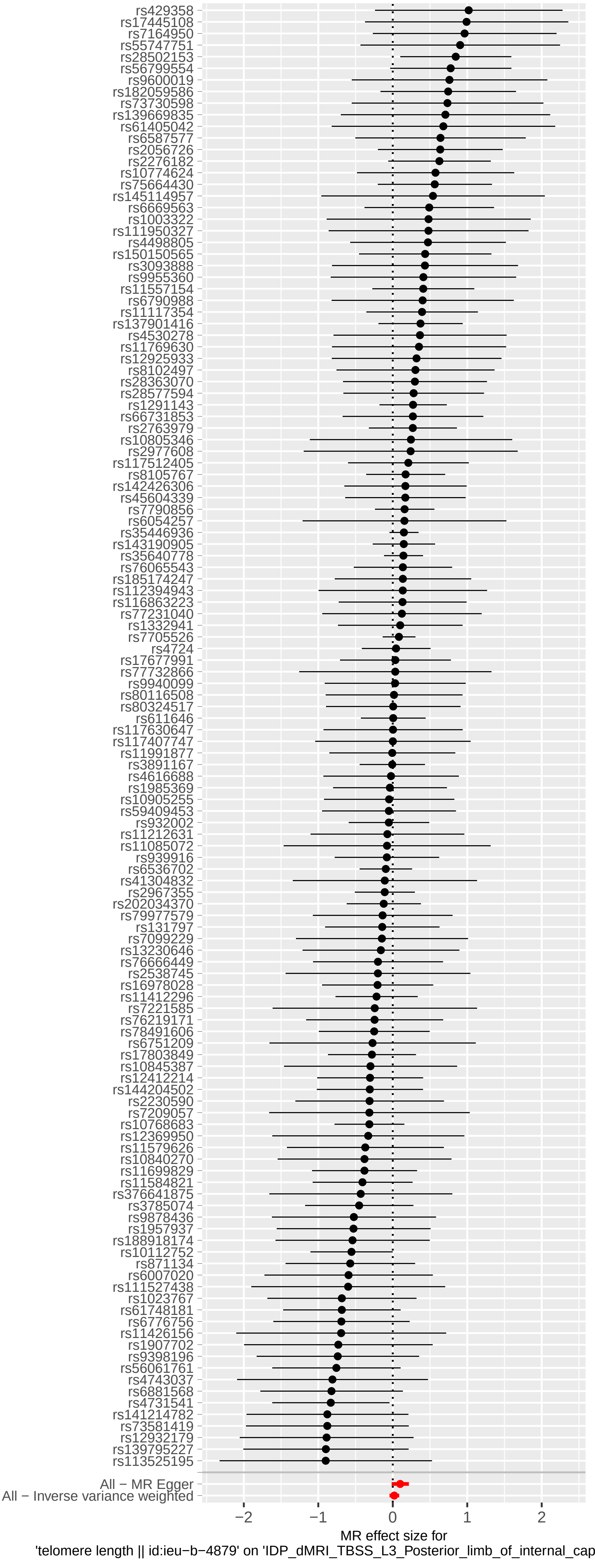
rs429358
rs17445108
rs7164950
rs55747751
rs28502153
rs56799554
rs9600019
rs182059586
rs73730598
rs139669835
rs61405042
rs6587577
rs2056726
rs2276182
rs10774624
rs75664430
rs145114957
rs6669563
rs1003322
rs111950327
rs4498805
rs150150565
rs3093888
rs9955360
rs11557154
rs6790988
rs11117354
rs137901416
rs4530278
rs11769630
rs12925933
rs8102497
rs28363070
rs28577594
rs1291143
rs66731853
rs2763979
rs10805346
rs2977608
rs117512405
rs8105767
rs142426306
rs45604339
rs7790856
rs6054257
rs35446936
rs143190905
rs35640778
rs76065543
rs185174247
rs112394943
rs116863223
rs77231040
rs1332941
rs7705526
rs4724
rs17677991
rs77732866
rs9940099
rs80116508
rs80324517
rs611646
rs117630647
rs117407747
rs11991877
rs3891167
rs4616688
rs1985369
rs10905255
rs59409453
rs932002
rs11212631
rs11085072
rs939916
rs6536702
rs41304832
rs2967355
rs202034370
rs79977579
rs131797
rs7099229
rs13230646
rs76666449
rs2538745
rs16978028
rs11412296
rs7221585
rs76219171
rs78491606
rs6751209
rs17803849
rs10845387
rs12412214
rs144204502
rs2230590
rs7209057
rs10768683
rs12369950
rs11579626
rs10840270
rs11699829
rs11584821
rs376641875
rs3785074
rs9878436
rs1957937
rs188918174
rs10112752
rs871134
rs6007020
rs111527438
rs1023767
rs61748181
rs6776756
rs11426156
rs1907702
rs9398196
rs56061761
rs4743037
rs6881568
rs4731541
rs141214782
rs73581419
rs12932179
rs139795227
rs113525195
All − MR Egger
All − Inverse variance weighted
−2
−1
0
1
2
MR effect size for
'telomere length || id:ieu−b−4879' on 'IDP_dMRI_TBSS_L3_Posterior_limb_of_internal_cap
